# Vertical Control of a Severe Hyperdivergent Skeletal Class II Malocclusion with Steep Posterior Occlusal Plane in a Camouflage Case

**DOI:** 10.3390/medicina58091217

**Published:** 2022-09-04

**Authors:** Yun Lu, Weihua Zhang, Bingjiao Zhao, Yuehua Liu

**Affiliations:** Department of Orthodontics, Shanghai Stomatological Hospital & School of Stomatology, Fudan University, Shanghai 200001, China; luyun@fudan.edu.cn (Y.L.); 17211370002@fudan.edu.cn (W.Z.); joyce_zhao_ortho@fudan.edu.cn (B.Z.)

**Keywords:** vertical control, high mandibular plane angle, steep posterior occlusal plane, open bite, miniscrew

## Abstract

Severe hyperdivergent skeletal Class II malocclusion may be ideally treated with orthognathic surgery in adult patients. Here, we report a camouflage treatment of a 23-year-old female patient. She was diagnosed with a skeletal Class II malocclusion with extreme high mandibular plane angle, retrusive mandible, steep posterior occlusal plane, anterior open bite, and severe overjet. The treatment plan included extraction of all second premolars and intrusion of the maxillary anterior teeth and mandibular posterior teeth using miniscrews. These contributed to an effective counterclockwise rotation of the mandible, decreased lower face height, and improvement in anterior overbite. This case report shows a vertical control strategy on severe hyperdivergent skeletal Class II malocclusions, which achieves well-controlled sagittal and vertical dimensions and a favorable facial appearance. The treatment and retention results were well balanced and aesthetically pleasing.

## 1. Introduction

Vertical control is of major importance in the treatment of hyperdivergent malocclusion characterized by a high mandibular plane angle and long face. The long face syndrome is formed by excessive vertical growth of the face with a backwardly rotated mandible, an increased lower face height, and a tendency of open bite in severe cases [1]. For adults characterized by skeletal class II malocclusion with an extreme high mandibular plane angle, orthognathic surgery is often required to reduce the vertical height and rotate the mandible counterclockwise [2]. However, many patients still have low acceptance of orthognathic surgery, and tend to choose nonsurgical methods such as orthodontic camouflage. For skeletal malocclusion, it is necessary to choose the appropriate treatment strategy according to the characteristics of different types of malocclusion.

In the treatment of skeletal Class II malocclusions, for patients with a retrognathic mandible and a convex facial profile, orthodontic camouflage can be performed with or without extractions using Class II elastics to coordinate the relationship between the maxilla and mandible [3]. However, Class II elastics generate some side effects, including the loss of mandibular anchorage and extrusion of the anterior and posterior teeth [4]. When this orthodontic approach is applied to high-angle patients, it induces a further increase in lower face height and a worse profile.

The emergence of miniscrews has undoubtedly enhanced the benefits of the vertical control of hyperdivergent patients [5]. For an anterior open bite, intrusion of the maxillary molars results in decreased facial height and counterclockwise rotation of the mandible [6,7]. However, a proportion of skeletal Class II malocclusions with a high mandibular plane angle have a steep posterior occlusal plane, which has been found to be closely related to short vertical height of the maxillary second molars [8] or has possibly contributed to an excessive height of the maxillary incisors [9]. Therefore, in such cases, decreasing the vertical dimension by intruding maxillary molars would seem unnecessary, while intrusion of the mandibular molars may be more indicated for achieving counterclockwise rotation of the mandible and to bring the cant of the occlusal plane closer to the norm. In severe cases, the vertical control of both mandibular posterior teeth and maxillary anterior teeth should be performed simultaneously.

This case report describes the camouflage treatment of skeletal Class II malocclusion with a retrusive mandible, an extremely high mandibular plane angle, an anterior open bite, a severe overjet, and a gummy smile. To rotate the mandible counterclockwise, the maxillary anterior teeth and mandibular posterior teeth were intruded using miniscrews. The treatment effectively provided profile improvement both vertically and sagittally.

## 2. Case Report

### 2.1. Diagnosis and Etiology

The female patient was 23 years old with a chief complaint of crooked teeth and protrusive mouth. She suffered from a lack of contact of her anterior teeth because of maxillomandibular sagittal discrepancy and tongue habit. She reported mouth breathing and snoring during sleep. There was no contributing medical history or significant TMJ symptoms.

**Figure 1:**
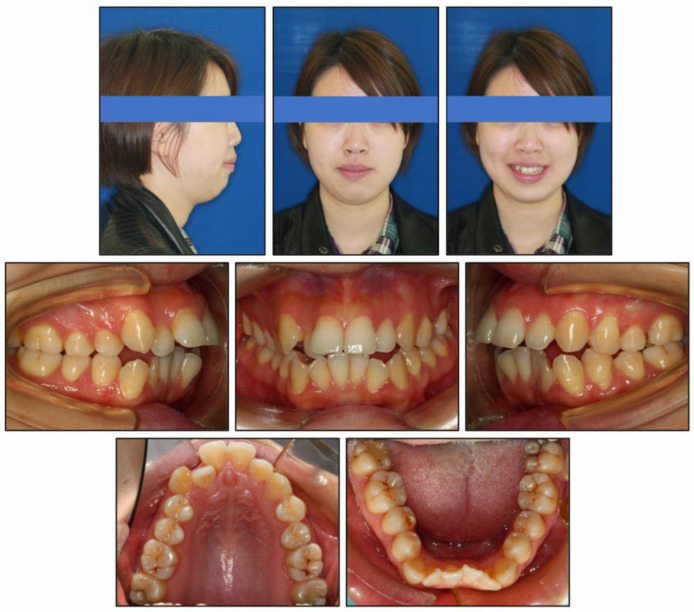
Pretreatment intraoral and facial photographs.

**Figure 2:**
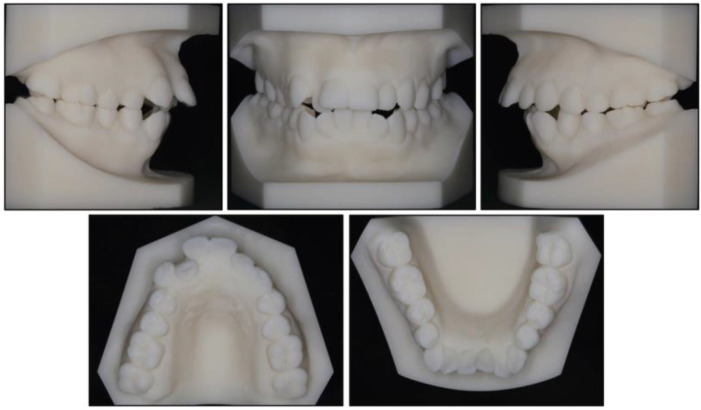
Pretreatment dental casts.

Photographs taken before treatment exhibited symmetrical facial structures, vertical growth excess, increased lower face height, and a convex profile. The convex profile and severe mentalis strain on lip closure were attributable to a retrognathic mandible and a high mandibular plane angle (Figure 1). The intraoral clinical examination (Figure 1) and pretreatment dental casts (Figure 2) showed that (1) in the sagittal direction, there was an Angle Class II malocclusion and a severe overjet; (2) in the vertical direction, there was a mild anterior open bite, a steep curve of Spee (3.5 mm), and an overgrowth of posterior and anterior alveolar bone; (3) in the horizontal direction, there was moderate maxillary crowding (4 mm), severe mandibular crowding (9 mm), and a V-shaped mandible; (4) in the Bolton analysis, the anterior ratio was 77.8% and the overall ratio was 90.5%; (5) periodontally, there was a slight recession in the gingivae of the mandibular anterior teeth and the mandibular left first premolar (LL4). Maxillary teeth and mandibular posterior teeth had no gingival inflammation or recession, and there was no increase in pocket depth. There was no complaint of pain or clicking in the TMJs.

**Figure 3:**
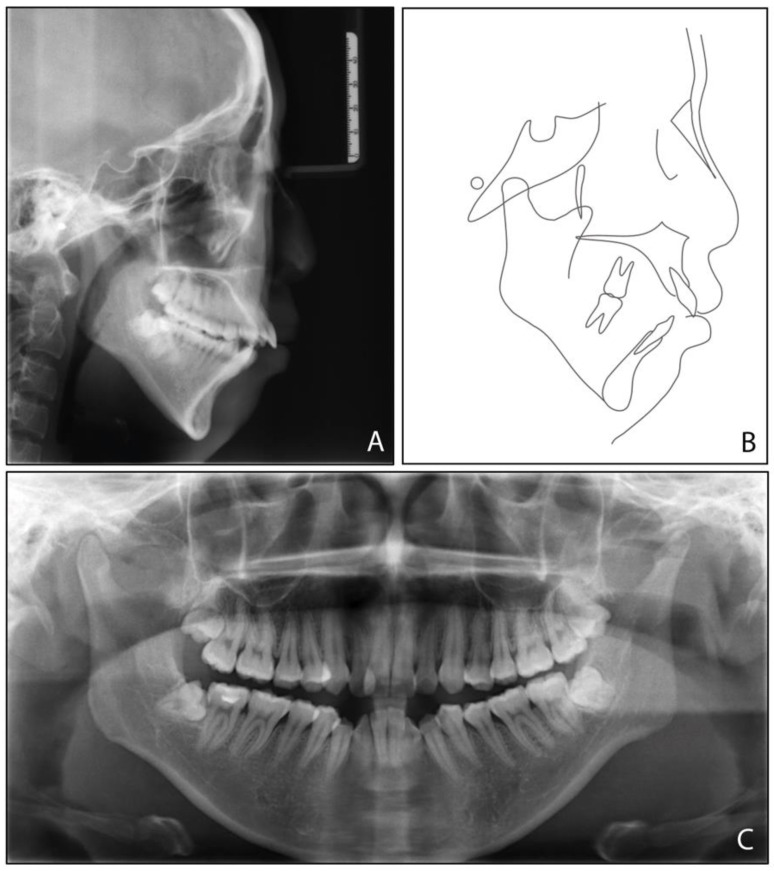
Pretreatment radiographs and tracing: (**A**) cephalometric radiograph; (**B**) cephalometric tracing; (**C**) panoramic radiograph.

The pretreatment panoramic radiograph (Figure 3) showed the presence of four third molars, among which the mandibular third molars were mesially impacted. The cephalometric analysis (Figure 3, Table 1) indicated a severe Class II skeletal relationship with mandibular retrusion (ANB, 8.4°, SNB, 71.9°), a steep mandibular plane angle (MP-SN, 55.8°), an excessive lower face height (81.1 mm), and a steep posterior occlusal plane (P-OP, 25.1°). The patient was thus diagnosed with an Angle Class II malocclusion, a mild anterior open bite, and a skeletal Class II condition caused by a high mandibular plane angle and retrognathic mandible.

### 2.2. Treatment Objectives

The main dental and profile treatment objectives for this patient were to (1) solve the crowding in both arches and widen the maxillary dental arch; (2) establish a functional occlusion by improving the anterior overjet and overbite; (3) improve occlusion by correcting the Class II molar relationship; (4) improve the convex profile by retracting maxillary anterior teeth; (5) decrease the vertical dimension and rotate the mandible counterclockwise by intruding mandibular posterior and maxillary anterior teeth; and (6) relieve the gummy smile.

### 2.3. Treatment Alternatives

We offered three treatment alternatives for our patient to consider. To correct the skeletal discrepancies, a combined surgical and orthodontic treatment was recommended: (1) extractions of maxillary second premolars and mandibular first premolars, (2) a LeFort I osteotomy to correct the overdevelopment of the maxilla in vertical dimension, (3) a bilateral sagittal split ramus osteotomy to achieve mandibular advancement and counterclockwise rotation, and (4) genioplasty to move the chin forward. This was considered an ideal option, which could correct the skeletal problems and achieve maximum improvement in facial appearance.

The second option was orthodontics alone to improve the convex profile and vertical overgrowth with sagittal and vertical control of the dentition. Miniscrew anchorage was used to retract the anterior teeth and intrude maxillary anterior and mandibular posterior segments. For a better vertical control effect, extracting second premolars is more conducive to intrusion of the mandibular molars due to the reduced burden on the posterior segment, compared to first premolar extraction. Moreover, there was no significant difference in the change in soft tissue profile after orthodontic treatment, regardless of the first or second premolar extraction [10]. Furthermore, miniscrews assisting movement of the anterior teeth can prevent loss of anchorage.

The third option was orthodontic treatment combined with genioplasty to improve the shape of the chin. After discussion with the patient, the first and third options were not adopted because she was reluctant to undergo surgery. Therefore, she consented to the second plan.

### 2.4. Treatment Progress

Extraction of the four second premolars and all third molars was performed before bonding. Subsequently, ceramic brackets (Inspire ICE, Ormco Corp, Orange, CA, USA) were placed in both arches.

**Figure 4:**
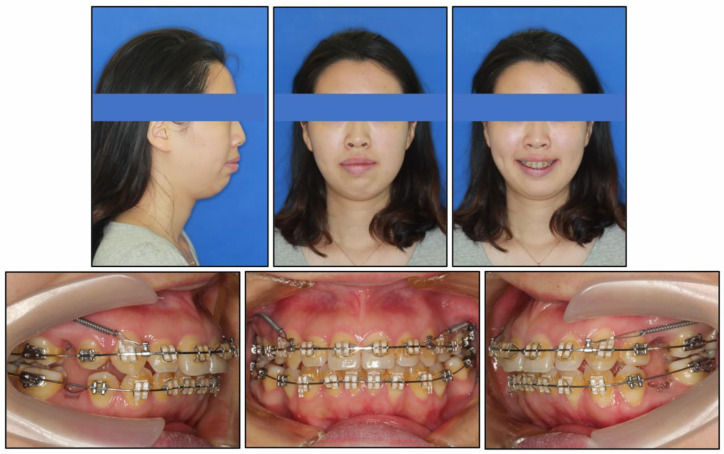
Eight months into treatment, effective alignment and leveling in both arches. Then, two miniscrews on the maxillary posterior segment were used to retract anterior teeth.

The archwire sequences were from 0.012-inch nickel titanium wire to 0.018 × 0.025-inch nickel titanium wire for alignment and leveling. Stainless steel archwires with the dimensions of 0.019 × 0.025 inches were used for space closing. Miniscrews (diameter, 1.6 mm; length, 11 mm; Cibei, Ningbo, China) were placed on the mesiobuccal alveolar bone of the second molar on both sides of the maxilla under local anesthesia. Nickel–titanium springs were used to retract the anterior teeth (Figure 4), as well as to improve the molar relationship.

**Figure 5:**
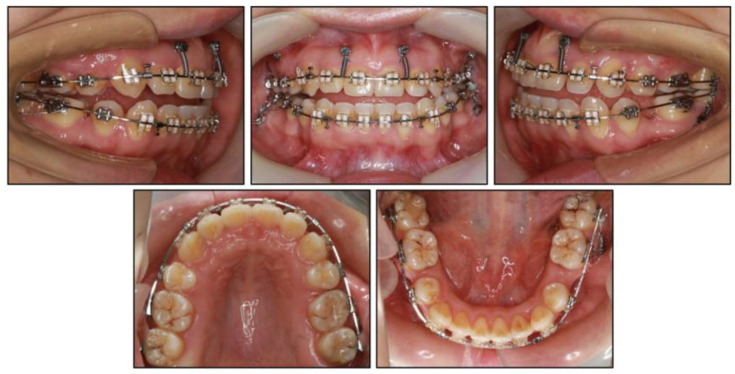
Twenty months into treatment, four miniscrews were used to intrude the maxillary anterior teeth and mandibular molars to achieve counterclockwise rotation of the mandible.

**Figure 6:**
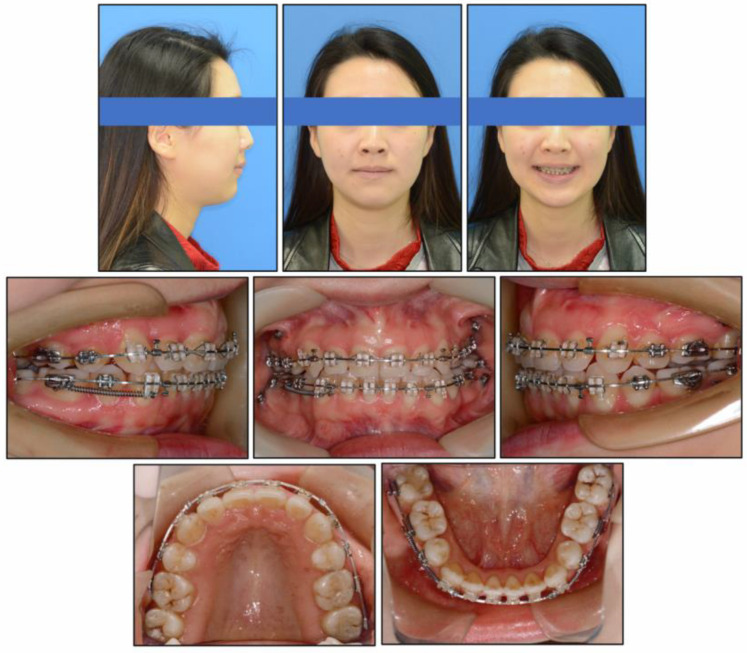
Twenty-seven months into treatment, 0.019 × 0.025-inch stainless steel archwires was used to close the residual spaces of both arches with spring to the miniscrews.

After 11 months, two miniscrews were placed on the buccal alveolar bone between the maxillary lateral incisor and the central incisor. The other two were inserted buccally into the alveolar bone between the mandibular first and second molars. During the intrusion stage, we observed a temporary anterior open bite (Figure 5). The intrusive force was approximately 100 gN. In order to prevent flaring of the mandibular posterior teeth, 25° of lingual root torque was applied to the 0.019 × 0.025-inch stainless steel archwires. After vertical control of the dentition, the anterior open bite was corrected (Figure 6). Interarch elastics were used to improve intercuspation in the fine adjustment phase.

The total orthodontic treatment period was 39 months. After removing the minisrcrews and appliances, a vacuum-formed retainer was required for retention in both the maxilla and mandible.

### 2.5. Treatment Results

**Figure 7:**
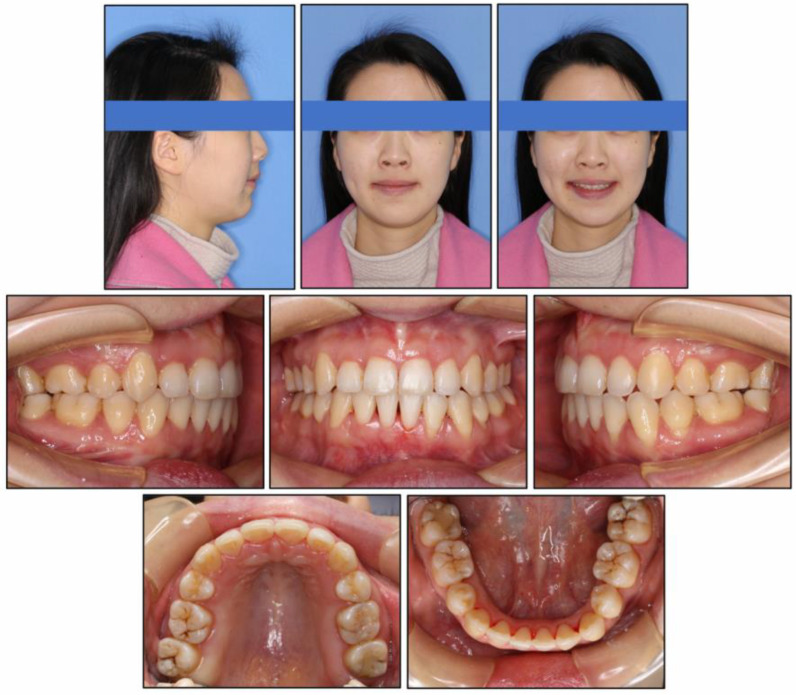
Post-treatment intraoral and facial photographs.

**Figure 8:**
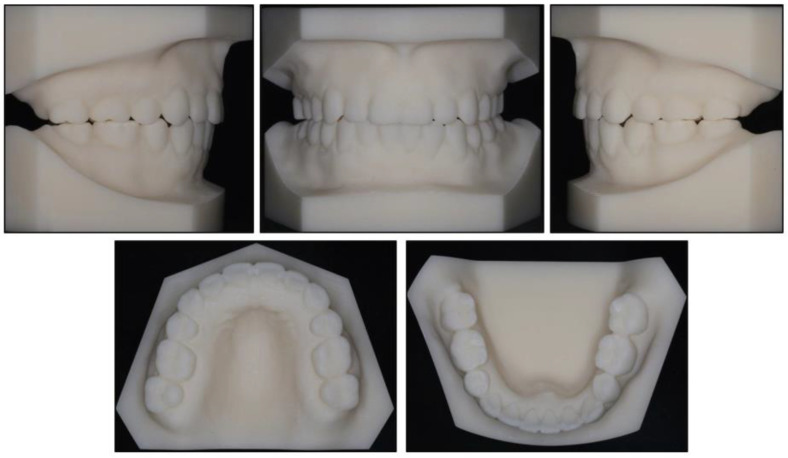
Post-treatment dental casts.

At the end of treatment, the patient’s facial profile and smile aesthetics were improved, with decreased lower face height. Her chin shape was improved significantly and the nose-lip-chin profile was more in harmony than before treatment. Intraoral photographs (Figure 7) and dental casts (Figure 8) showed well-aligned teeth, a satisfactory overbite and overjet, a gentle posterior occlusal plane, and a Class I molar relationship.

**Figure 9:**
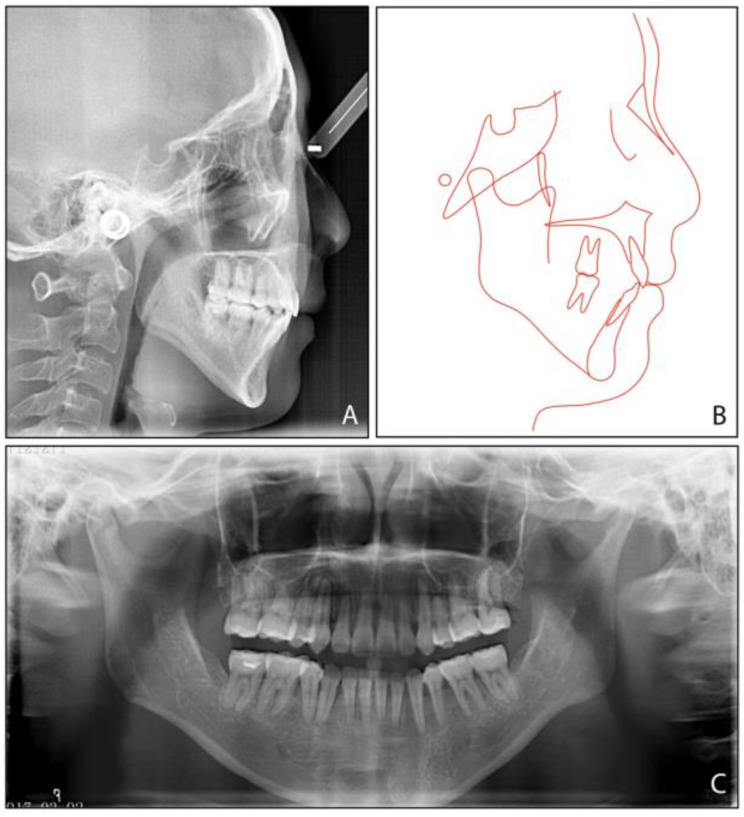
Post-treatment radiographs and tracing: (**A**) cephalometric radiograph; (**B**) cephalometric tracing; (**C**) panoramic radiograph.

**Figure 10:**
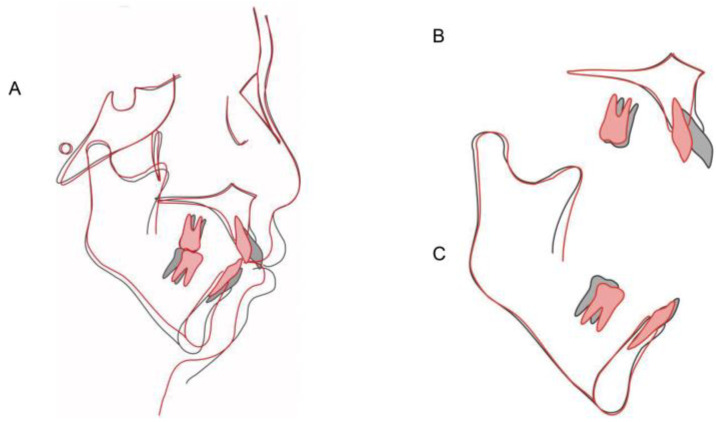
Cephalometric superimpositions of pretreatment (black) and post-treatment (red): (**A**) SN plane; (**B**) maxillary plane; (**C**) mandibular plane.

The final panoramic radiograph (Figure 9) showed mild root resorption and maxillary right canine periodontal space widening. There were no obvious signs of bone or apical root resorption compared to the pretreatment panoramic view. According to the cephalometric analysis (Figure 9 and Figure 10, Table 1), changes in appearance included the counterclockwise rotation of the mandibular plane (MP-SN had been reduced by 2.9°) and decreases in lower face height (reduced by 5.7 mm) and sagittal discrepancy (ANB had been reduced by 2.6°). Dental changes included the retraction of the maxillary and mandibular incisors, intrusion of both the maxillary incisors and mandibular molars (U1-PP and L6-MP had been reduced by 6.3 mm and 4.5 mm, respectively), and the amount of posterior occlusal plane (P-OP) rotation was 8.9°.

**Figure 11:**
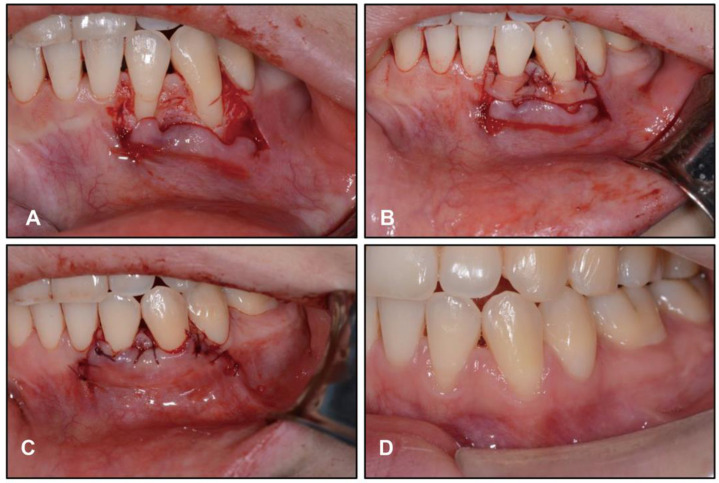
Twelve months after orthodontic treatment, intraoral photographs showed the mucogingival surgery procedure of left lower lateral incisor and left lower canine: (**A**) flap detachment beyond the mucogingival junction; (**B**) tissue graft fixed with resorbable sutures; (**C**) the flap fixed with sling sutures at the papillae; (**D**) two months after surgery.

**Figure 12:**
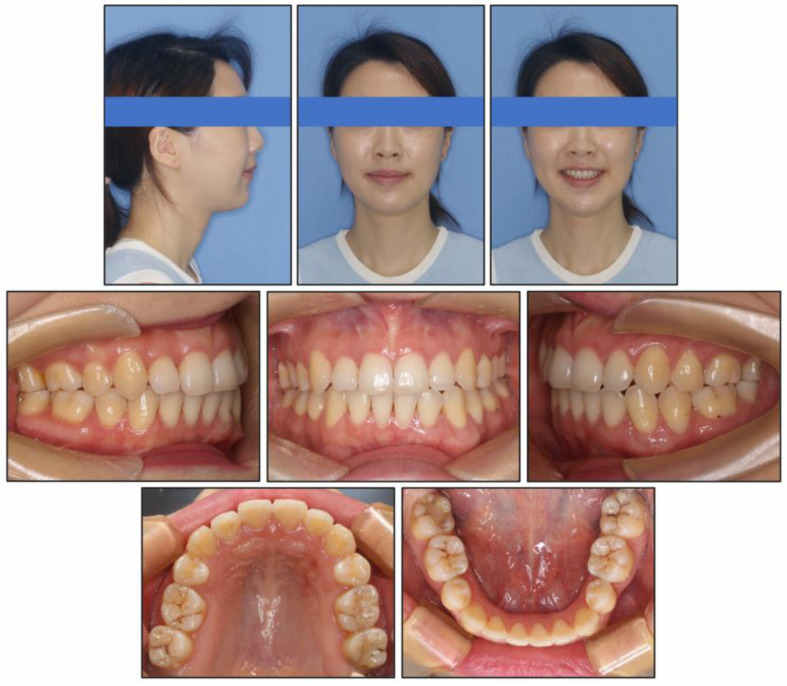
Follow-up intraoral and facial photographs at two years six months.

The post-treatment intraoral photographs showed gingival recession in LL2 and LL3; therefore, mucogingival surgery was performed by a periodontist after 12 months of retention (Figure 11). The patient was treated with root coverage procedure of a coronal advanced flap with a subepithelial connective tissue graft. At her 2 years and 6 months follow-up, our patient was maintaining excellent oral hygiene and stability of the hard and soft tissues. (Figure 12). Moreover, no TMJ problems were found during either treatment or retention.

## 3. Discussion

Skeletal Class II malocclusion with high mandibular plane angle has always been a challenging condition for orthodontics, for both its sagittal and vertical discrepancies. The vertical overgrowth has heredity as the major determinant, but environmental factors such as sleep-disordered breathing symptoms [11] and mouth breathing caused by adenoid and tonsillar hypertrophy [12] act as secondary causes of the long face and mandibular retrusion. Mouth breathers tended to have a vertical growth pattern with high mandibular plane angle, downward and backward rotation of the mandible, an increase in total and lower anterior facial height, and a decrease in posterior facial height [13]. For these patients, if excessive vertical development of the lower face is accompanied by increased molar eruption, anterior open bite may occur [14]. Moreover, the increase in vertical height reinforces the tension of the labial muscles, distorting the chin.

Orthognathic surgery is recommended in the treatment of hyperdivergent skeletal Class II malocclusion, but many patients decline surgery because of anxiety and the risk of unwanted side effects. Several treatment approaches have been proposed to control vertical dimensions in hyperdivergent patients, including cervical or high-pull headgear [15], chincup [16], and vertical control of molars [17,18]. Recently, miniscrews have been widely used in orthodontic clinics, and many studies have shown promising results in vertical control [5,19,20,21]. Clinical applications of miniscrews have broadened the scope of non-surgical orthodontic treatments [22]. For patients with hyperdivergent skeletal Class II malocclusion, miniscrews can achieve significant intrusion of both anterior and posterior teeth, providing space for counterclockwise rotation of the mandible which causes the chin to move forward and relieves the tension on the mentalis through sagittal and vertical changes [23]. Miniscrews are also used as sagittal anchorage for retracting anterior teeth. In our patient, we initially implanted miniscrews into the maxilla as maximum anchorage to correct the Class II molar relationship and excess overjet. Next, vertical control was achieved by intruding the mandibular molars with miniscrews because of the extremely high mandibular plane angle (55.8°).

**Figure 13:**
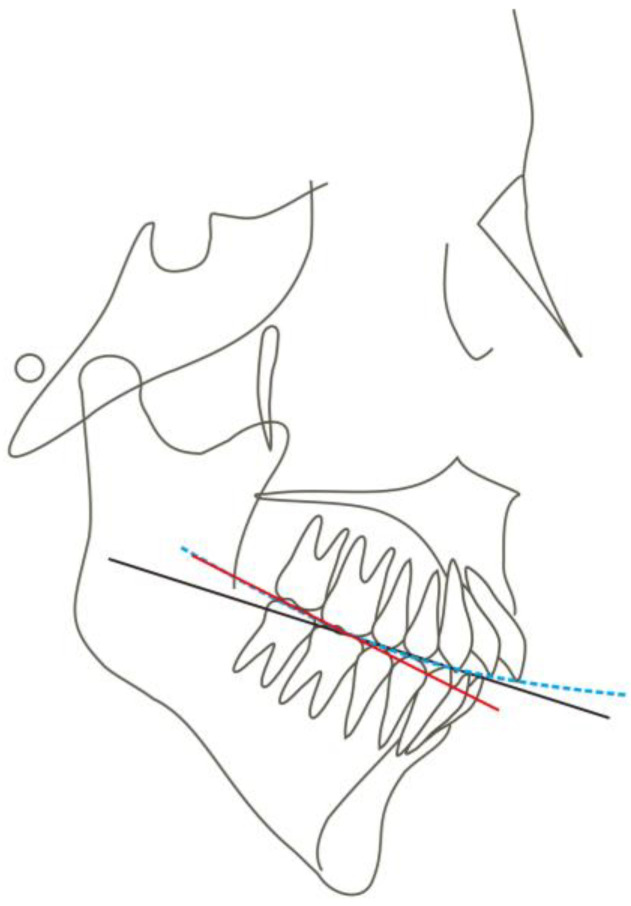
A schematic of skeletal Class II malocclusion with a steep posterior occlusal plane. The black solid line indicates the conventional occlusal plane, blue dotted line indicates the curve of Spee, red solid line indicates the posterior occlusal plane.

**Figure 14:**
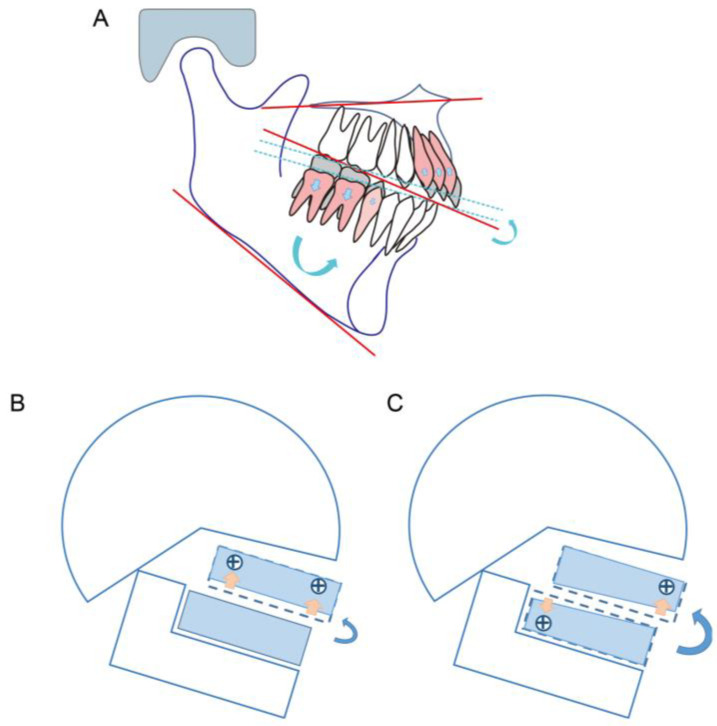
A schematic of mandibular rotation: (**A**) the achievement of occlusal rotation by correcting the excessive eruption of maxillary incisors and mandibular molars; (**B**,**C**) two strategies in vertical control. Intrusion of maxillary anterior teeth and mandibular posterior teeth (**C**) can provide more counterclockwise rotation of mandible plane than maxillary anterior teeth and maxillary posterior teeth (**B**) in patients with a steep posterior occlusal plane.

For long-face patients with anterior open bite, intrusion of the posterior teeth results in counterclockwise mandibular rotation and increased overbite [24]. Although there are many case reports addressing patients with anterior open bite by intruding maxillary molars, there are few reports of intruding mandibular molars. We have postulated two reasons: Firstly, intruding mandibular molars is more difficult than maxillary molars because the bone density of the posterior mandible is higher than that of the posterior maxilla [25]. Secondly, the maxillary molars are required to exert an intrusive force on both buccal and palatal sides, while it is difficult to implant miniscrews on the lingual side of mandibular molars. Indeed, intruding maxillary molars is a common choice. However, studies on the treatment effects on these patients pay more attention to the change in the mandibular plane angle and less on the change in the occlusal plane. In some cases, the occlusal plane does not reflect the true occlusion because it does not consider the height of the second molar. If we draw in a conventional way, the occlusal plane of a patient with a deep curve of Spee may be normal and flat. However, if the occlusal surface of the first molar and the second molar is connected, the canting of the posterior occlusal plane will be steep (Figure 13). Fushima [8] found that skeletal Class II patients with an extremely high mandibular plane angle had a steep posterior occlusal plane, which was found to be closely related to a short vertical height of the maxillary second molars. It is crucial to flatten the steep posterior occlusal plane by controlling the molar vertical dimension. The importance of molar height to occlusal plane variation has been recognized. Arriola et al. [26] showed that skeletal open-bite Class II patients had greater mandibular molar heights. One possible reason is that Class II patients have a small and retruded mandible, lacking space for the erupting third molars, which could tend to extrude the second molars and increase the occlusal plane angle. Therefore, the intrusion of the mandibular molars may be more indicated in such cases to achieve counterclockwise rotation of the mandible and to make the cant of the occlusal plane closer to the norm. On the other hand, Ye et al. [9] reported that patients with hyperdivergent skeletal Class II malocclusions had steep occlusal planes with excessive height of the incisors. These findings suggest that treatment for such patients should be aimed primarily at correcting the excessive eruption of maxillary incisors and mandibular molars. In the present case, we successfully achieved improvement in the open bite and counterclockwise rotation of the mandible by intruding mandibular molars and maxillary anterior teeth, which may provide more rotation of the posterior occlusal plane than intruding both anterior and posterior teeth of maxillary (Figure 14).

When intruding the mandibular molars, buccal miniscrews provide both a buccal force and an intrusive force, the latter can cause buccal inclination of molars. To overcome this problem, an archwire with 25° lingual crown torque was applied. The mandibular molars did not appear buccally inclined during the treatment, which indicated that the above method is effective. Furthermore, a lingual arch, as a multipurpose appliance, can also be used to prevent the molars from buccal inclination [27]. Compared with increasing the lingual crown torque of the archwires, the lingual arch may provide greater anchorage to the molars, but more discomfort to the patient [28].

In the treatment of vertical control with miniscrews, some complications such as root resorption, soft-tissue irritation, and relapse need to be taken into account when intruding teeth [29]. Thus, intrusive force should be light and continuous to reduce the risk of root resorption. The loading force we used was about 100 gN per side. Moderate gingival recession after treatment was noticeable in the left mandibular lateral incisor and canine (Figure 11) for which mucogingival surgery was performed to improve the appearance. During retention, the patient was urged to perform masticatory exercises to minimize vertical relapse [30]. As shown in Figure 12, stability of the treatment result was fine in further follow-up.

## 4. Conclusions

This case showed a vertical control strategy on a severe hyperdivergent skeletal Class II malocclusion by miniscrew-assisted intrusion of both the maxillary anterior teeth and mandibular posterior teeth, which achieved an effective counterclockwise rotation of the mandible and a favorable facial appearance. Patients with the following characteristics can be treated with this strategy if they refuse orthognathic surgery: long lower face height, a high-angle mandibular plane with a steep posterior occlusal plane, excessive overjet, anterior open bite, and deep overbite with gummy smile.

## Figures and Tables

**Table 1:** Cephalometric measurements.

Measurement	Normal Mean ± SD	Pretreatment	Posttreatment	Difference
SNA (°)	83.1 ± 2.7	80.3	79.7	−0.6
SNB (°)	80.3 ± 2.6	71.9	73.9	2
ANB (°)	2.7 ± 1.8	8.4	5.8	−2.6
UI-SN (°)	103.4 ± 5.5	106.9	91.9	−15
LI-MP (°)	96.3 ± 5.4	97.2	90.8	−6.4
UI-LI (°)	129.1 ± 7.1	102.7	129.1	26.4
MP-SN (°)	32.6 ± 6.9	55.8	52.9	−2.9
MP-FH (°)	25.5 ± 4.8	45.6	42.8	−2.8
Wits (mm)	−1 ± 1	5.8	2.8	−3
A-OP (°)	10 ± 3.58	12.2	14.5	2.3
P-OP (°)	14.9 ± 3.85	25.1	16.2	−8.9
U1-PP (mm)	28 ± 1.6	32.9	26.6	−6.3
U6-PP (mm)	23 ± 1	24.4	19.3	−5.1
L1-MP (mm)	40.8 ± 1.8	44.1	39.3	−4.8
L6-MP (mm)	31.1 ± 1.9	33.4	28.9	−4.5
Palatal-OP (°)	10 ± 4	17	15.7	−1.3
Upper Face Height (mm)	50 ± 2.5	56.4	55.8	−0.6
Lower Face Height (mm)	65 ± 4.5	81.1	75.4	−5.7
Anterior Face Height (mm)	115 ± 5.5	135.9	130.8	−5.1
Posterior Face Height (mm)	45 ± 5	75.9	73.2	−2.7
P-A Face Height (%)	65 ± 4	55.8	56	0.2
Y-Axis (°)	67 ± 5.5	84.7°	83.1	−1.6
UL-E (mm)	−1.6 ± 1.5	5	−1	−6
LL-E (mm)	−0.2 ± 1.9	7	−1.3	−8.3

SN, sella-nasion plane; MP, mandibular plane; FH, Frankfort horizontal plane; OP, occlusal plane; A-OP, anterior occlusal plane; P-OP, posterior occlusal plane; PP, palatal plane.

## Data Availability

All experimental data supporting the results of this study are available from Shanghai Stomatological Hospital (China).

## References

[1] Merville L.C., Diner P.A. (1987). Long face: New proposals for taxonomy, diagnosis, treatment. J. Craniomaxillofac. Surg..

[2] Proffit W.R., White R.J. (1990). Who needs surgical-orthodontic treatment?. Int. J. Adult Orthodon. Orthognath. Surg..

[3] Janson G., Sathler R., Fernandes T.M., Branco N.C., Freitas M.R. (2013). Correction of Class II malocclusion with Class II elastics: A systematic review. Am. J. Orthod. Dentofac. Orthop..

[4] Ellen E.K., Schneider B.J., Sellke T. (1998). A comparative study of anchorage in bioprogressive versus standard edgewise treatment in Class II correction with intermaxillary elastic force. Am. J. Orthod. Dentofac. Orthop..

[5] Lee J., Miyazawa K., Tabuchi M., Kawaguchi M., Shibata M., Goto S. (2013). Midpalatal miniscrews and high-pull headgear for anteroposterior and vertical anchorage control: Cephalometric comparisons of treatment changes. Am. J. Orthod. Dentofac. Orthop..

[6] Rachala M.R., Harikrishnan P. (2010). Management of severe anterior open-bite in an adult patient using miniscrews as skeletal anchorage. Int. J. Orthod. Milwaukee.

[7] Yanagita T., Adachi R., Kamioka H., Yamashiro T. (2013). Severe open bite due to traumatic condylar fractures treated nonsurgically with implanted miniscrew anchorage. Am. J. Orthod. Dentofac. Orthop..

[8] Fushima K., Kitamura Y., Mita H., Sato S., Suzuki Y., Kim Y.H. (1996). Department of Orthodontics, Kanagawa Dental College, Yokosuka, Kanagawa, Japan. Significance of the cant of the posterior occlusal plane in class II division 1 malocclusions. Eur. J. Orthod..

[9] Ye R., Li Y., Li X., Li J., Wang J., Zhao S., Zhao Z. (2013). Occlusal plane canting reduction accompanies mandibular counterclockwise rotation in camouflaging treatment of hyperdivergent skeletal Class II malocclusion. Angle Orthod..

[10] Omar Z., Short L., Banting D.W., Saltaji H. (2018). Profile changes following extraction orthodontic treatment: A comparison of first versus second premolar extraction. Int. Orthod..

[11] Huynh N.T., Morton P.D., Rompre P.H., Papadakis A., Remise C. (2011). Associations between sleep-disordered breathing symptoms and facial and dental morphometry, assessed with screening examinations. Am. J. Orthod. Dentofac. Orthop..

[12] Acharya S.S., Mali L., Sinha A., Nanda S.B. (2020). Effect of Naso-respiratory Obstruction with Mouth Breathing on Dentofacial and Craniofacial Development. Orthod. J. Nepal.

[13] Zheng W., Zhang X., Dong J., He J. (2020). Facial morphological characteristics of mouth breathers vs. nasal breathers: A systematic review and meta-analysis of lateral cephalometric data. Exp. Ther. Med..

[14] Kuitert R., Beckmann S., van Loenen M., Tuinzing B., Zentner A. (2006). Dentoalveolar compensation in subjects with vertical skeletal dysplasia. Am. J. Orthod. Dentofac. Orthop..

[15] Zervas E.D., Galang-Boquiren M.T., Obrez A., Costa Viana M.G., Oppermann N., Sanchez F., Romero E.G., Kusnoto B. (2016). Change in the vertical dimension of Class II Division 1 patients after use of cervical or high-pull headgear. Am. J. Orthod. Dentofac. Orthop..

[16] Buschang P.H., Sankey W., English J.P. (2002). Early treatment of hyperdivergent open-bitemalocclusions. Semin. Orthod..

[17] Deberardinis M., Stretesky T., Sinha P., Nanda R.S. (2000). Evaluation of the vertical holding appliance in treatment of high-angle patients. Am. J. Orthod. Dentofac. Orthop..

[18] Villalobos F.J., Sinha P.K., Nanda R.S. (2000). Longitudinal assessment of vertical and sagittal control in the mandibular arch by the mandibular fixed lingual arch. Am. J. Orthod. Dentofac. Orthop..

[19] Wang X.D., Zhang J.N., Liu D.W., Lei F.F., Zhou Y.H. (2016). Nonsurgical correction of a severe anterior deep overbite accompanied by a gummy smile and posterior scissor bite using a miniscrew-assisted straight-wire technique in an adult high-angle case. Korean J. Orthod..

[20] Wang X.D., Zhang J.N., Liu D.W., Lei F.F., Liu W.T., Song Y., Zhou Y.H. (2017). Nonsurgical correction using miniscrew-assisted vertical control of a severe high angle with mandibular retrusion and gummy smile in an adult. Am. J. Orthod. Dentofac. Orthop..

[21] Jung M.H. (2019). Vertical control of a Class II deep bite malocclusion with the use of orthodontic mini-implants. Am. J. Orthod. Dentofac. Orthop..

[22] Choi S.H., Jeon J.Y., Lee K.J., Hwang C.J. (2021). Clinical applications of miniscrews that broaden the scope of non-surgical orthodontic treatment. Orthod. Craniofac. Res..

[23] Kim K., Choy K., Park Y.C., Han S.Y., Jung H., Choi Y.J. (2018). Prediction of mandibular movement and its center of rotation for nonsurgical correction of anterior open bite via maxillary molar intrusion. Angle Orthod..

[24] Umemori M., Sugawara J., Mitani H., Nagasaka H., Kawamura H. (1999). Skeletal anchorage system for open-bite correction. Am. J. Orthod. Dentofac. Orthop..

[25] Turkyilmaz I., Tozum T.F., Tumer C. (2007). Bone density assessments of oral implant sites using computerized tomography. J. Oral. Rehabil..

[26] Arriola-Guillen L.E., Flores-Mir C. (2014). Molar heights and incisor inclinations in adults with Class II and Class III skeletal open-bite malocclusions. Am. J. Orthod. Dentofac. Orthop..

[27] Sunny S., Joseph D.P., Mathew N., Rajan R.S., Kurian E. (2017). Three-Dimensional Control on Lingually Rolled in Molars using a 3D Lingual Arch. J. Clin. Diagn. Res..

[28] Sharma H.S. (2002). Orthodontic Anchorage Enhancement with Lingual Arch. Med. J. Armed Forces India.

[29] Kravitz N.D., Kusnoto B., Tsay T.P., Hohlt W.F. (2007). The use of temporary anchorage devices for molar intrusion. J. Am. Dent. Assoc..

[30] Parks L.R., Buschang P.H., Alexander R.A., Dechow P., Rossouw P.E. (2007). Masticatory exercise as an adjunctive treatment for hyperdivergent patients. Angle Orthod..

